# Supplementary material for: Site-dependent reactivity of MoS2 nanoparticles in hydrodesulfurization of thiophene
Source: Nat Commun. 2020 Aug 31;11:4369. doi: 10.1038/s41467-020-18183-4 (PMC7459117; doi:10.1038/s41467-020-18183-4)
Supplement: Supplementary file 1 — Supplementary Information [file 41467_2020_18183_MOESM1_ESM.pdf]

SUPPLEMENTARY INFORMATION

Site-dependent Reactivity of MoS_2 Nanoparticles in Hydrodesulfurization of Thiophene

Norberto Salazar[1,†], Srinivas Rangarajan[2,‡], Jonathan Rodríguez Fernández[1],

Manos Mavrikakis[2,*], Jeppe V. Lauritsen[1,*]

Supplementary Figure 1

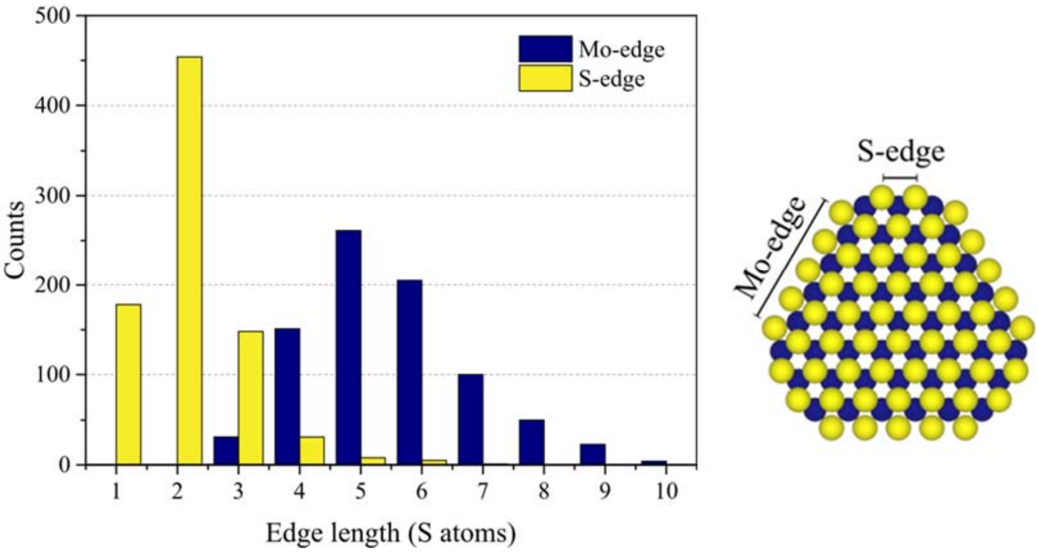

Distribution of S-edges and Mo-edge lengths reflecting the ensemble of synthesized r-MoS_2 nanoparticles. Left: Length distribution for the Mo and S-edges of MoS_2 nanoparticles. Right: Average MoS_2 nanoparticle morphology based on the total area of Mo and S-edges. A Mo edge length is denoted by its number of S atoms, e.g. as Mo-5S for an edge with 5 S atoms. After the reductive conditions were applied, MoS_2 nanoparticles can adopt multiple shape conformations, all governed by the MoS_2 symmetry. As expected, the newly-formed S-edges show shorter atomic lengths with respect to the Mo-edges, whose lengths can span up to about ten atoms. It is noted that the S-edges of two atoms are predominant for all the experimental series, while Mo-edges between four to six atoms are very frequent as shown in Supplementary Figure 1. In order to obtain the lowest possible statistical error, the analysis of the coverage of individual S vacancies at the Mo-edge after each experiment was carried out for the four (Mo-4S), five (Mo-5S) and six (Mo-6S) S-atoms-long Mo-edges, which were the most representative Mo-edges as shown above.

Supplementary Figure 2

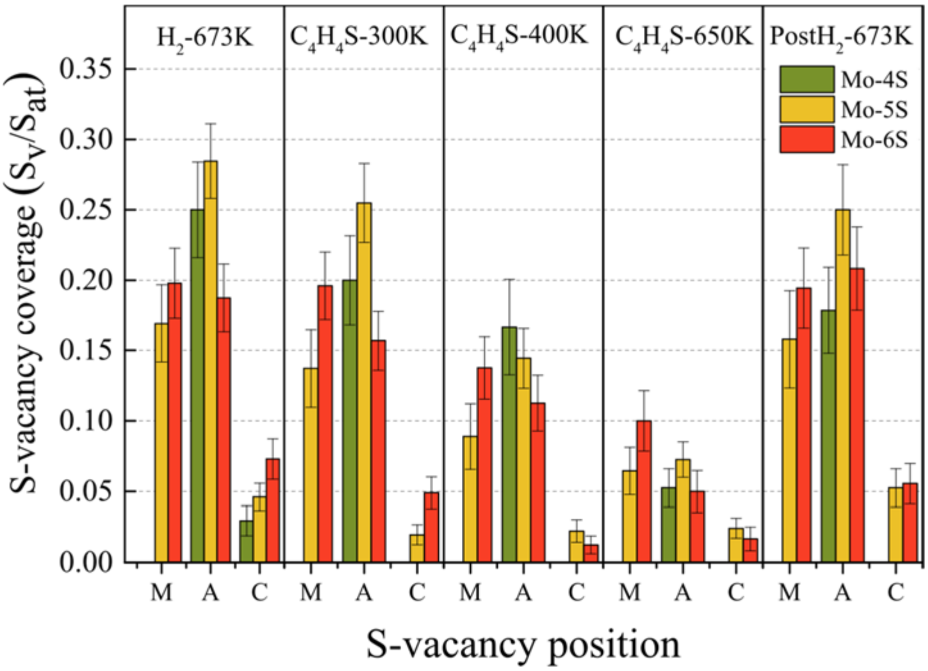

Statistical break-down of data for sulfur vacancy coverage for Mo-edges of length Mo-4S, Mo-5S and Mo-6S as a function of the S vacancy position at M, A and C for MoS_2 nanoparticles exposed to H_2 at 673 K, thiophene at 300 K, 400 K and 650 K and post-annealed in H_2 at 673 K. In order to investigate the adsorption behavior as a function of the Mo-edge length, a more detailed study of the occupancy of vacancies at C, A and M sites was performed. The S vacancy fraction per each Mo-edge at different temperatures is depicted in Supplementary Figure 2. Error bars reflect the statistical uncertainty based on the standard error $\sqrt{N}$, where N is the number of observations. At 300 K in thiophene, numerous changes are observed in the evolution of the vacancy fraction, especially the decline of vacancies at position C for all the edges represented in the graph. In the case of the shortest edges (Mo-4S), the vacancies at the corner sites disappear completely whereas for the longest edges (Mo-6S) the decrease is gradual. Surprisingly, for Mo-6S edges the vacancy at position M does not decrease and instead it keeps the same coverage. However, for position A, the decrease of the vacancy fraction is significant for all the lengths. At 400 K, for the most representative edge namely the Mo-5S, a pronounced change occurs at position A, where the coverage varies from 0.25 to 0.14, showing that these sites are preferable for the adsorption of the molecule. In the case of position C, the coverage is kept constant at much lower values. In addition, positions A and M for the Mo-6S edge change by 0.04 and 0.06, respectively, while coverage reduction for the corners sites is also noted. A temperature increase to 650 K during the exposure of the molecules, again leads to a tendency towards decreased number of vacancies. Vacancies could be generated again in the reductive H_2 treatment as it is clearly shown in the graph plot as PostH_2-673K. In fact, the largest formation of uncoordinated sites occurs at position A for the Mo-5S and Mo-6S edges where the increase in the S vacancy coverage is of approximately 0.17.

Supplementary Figure 3

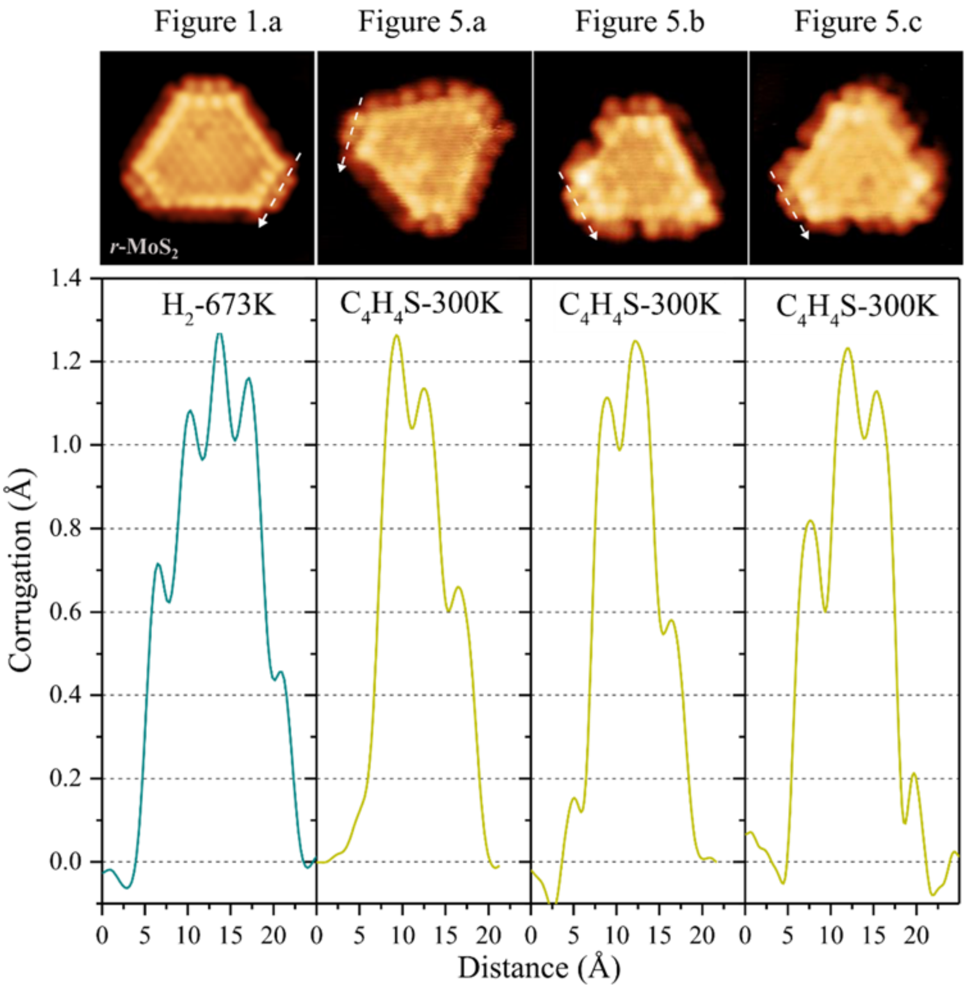

Data for the S edge: Comparison of line scans performed on the S-edges of r-MoS_2 and for the subsequent thiophene exposure series at 300K. Note that an S-edge with a 3 S atom edge length is present in the first image whereas S edges with two S atoms are present in the three subsequent images. The ~0.1Å height modulation between neighboring protrusions on the S edge is associated with the pairwise splitting and formation of S_2 dimers on the S edge, as illustrated in Figure 2b (side view). We note, however, that the experiment suggests a dimer located in the middle whereas the model in Figure 2b shows the inverse. This may be due to slight energy variations, possibly from interaction with the Au substrate.

Supplementary Figure 4

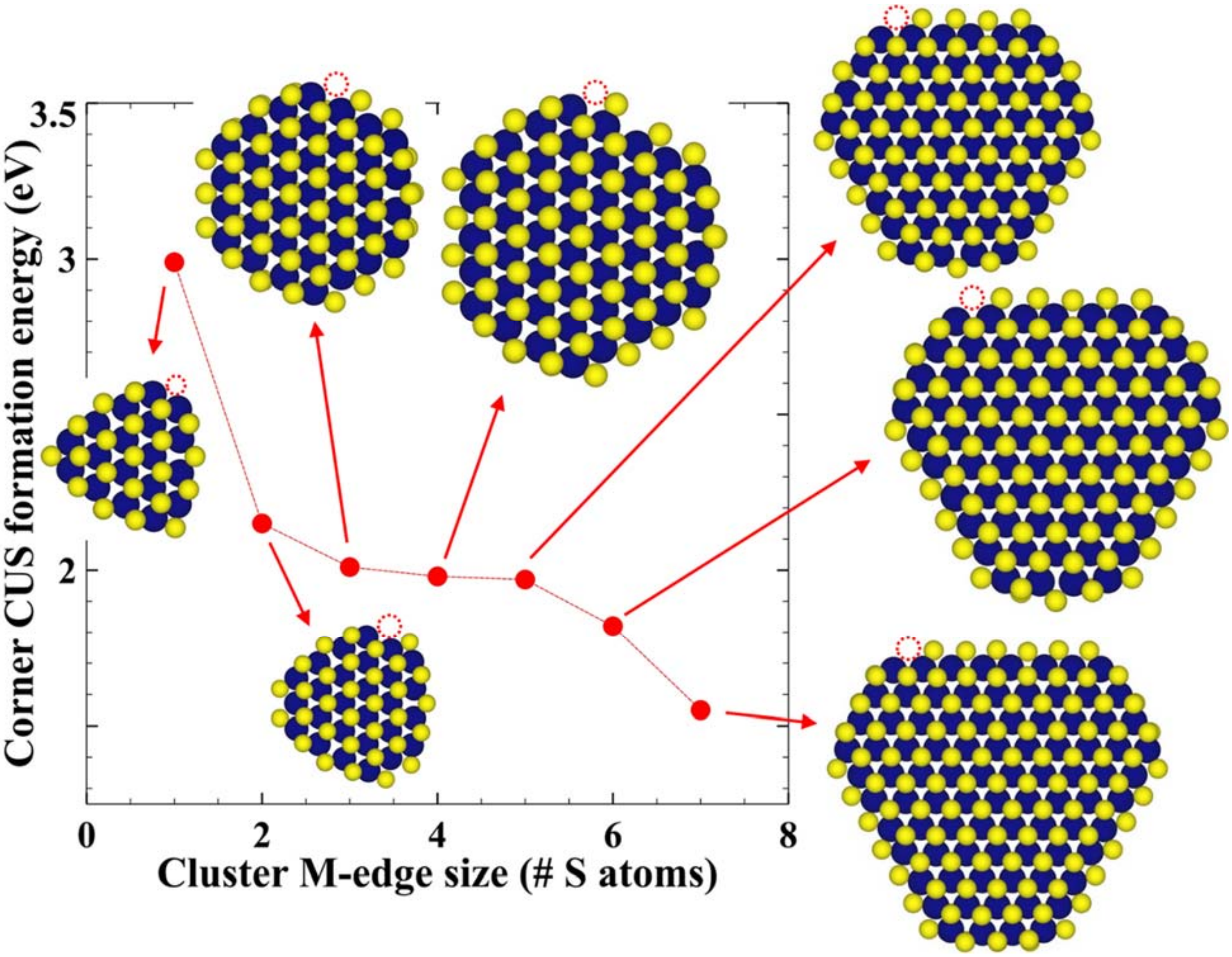

.

Vacancy formation energy on the corner site for different particle sizes (Mo-edge size). The graph expands on Figure 2 in the main text and shows specific structures and the location of the CUS sites.. Specific nanoparticles are also shown. Vacancy locations are marked with a dashed red circle. Alternative stable adsorption structures of thiophene are shown in Supplementary Table 1 below. The binding energy (BE) and notes for each structure are also given. These structures are shown purely as illustration since many other local minima exist. In particular, these structures show that dimerization of S as well as displacement to the top site can occur together as seen in STM structures (5b and 5c in the main text). Further, we also note that adsorption structures where thiophene is located between typical A and M locations (designated A-M) are also energetically feasible although not the most stable ones. Since thiophene adsorption is activated, some of the observed STM configurations may be kinetically trapped even if they are not the most stable.

Supplementary Table 1

Alternative stable adsorption structures of thiophene on the Mo-edge of MoS_2 particles. Both top and edge views are shown. Red circles indicate the formation of dimers (shown in edge views).

**(i) BE = -0.50 eV**<br>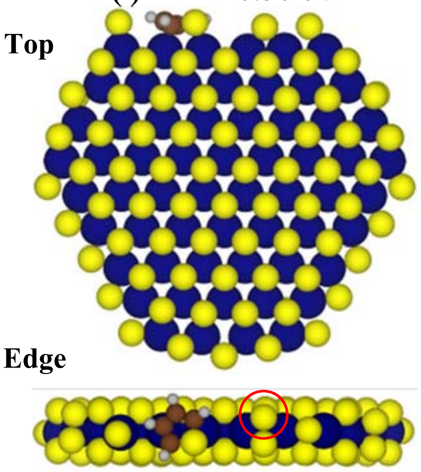<br><br>**Notes: Alternative structure of thiophene adsorption on the A-M sites with S-dimerization and the displacement of corner S to create space for thiophene to bind.**	**(ii) BE = -0.56 eV**<br>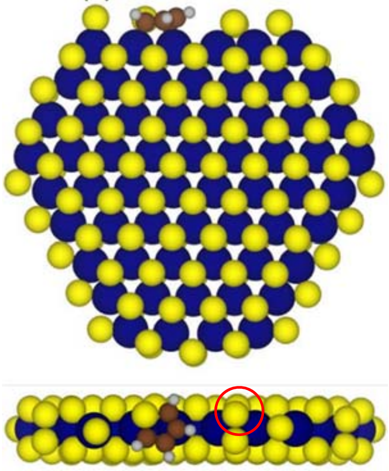<br>**Notes: Alternative structure of thiophene on the A-M sites with S-dimerization and the displacement of corner S to create space for thiophene to bind.**
**(iii) BE = -0.37 eV**<br>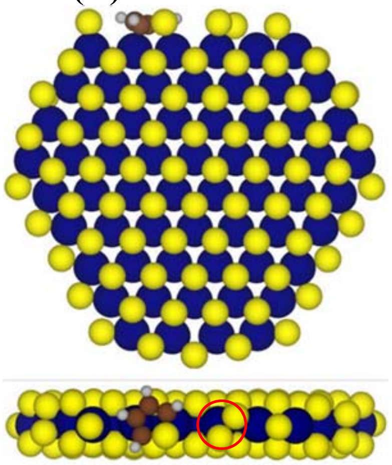<br>**Notes: Alternative structure of thiophene on the A-M sites with S-dimerization and the displacement of corner S. The dimer has not fully formed (indicating this is potentially a metastable or local minima) and the S of thiophene points to the dimer.**	**(iv) BE = -0.69 eV**<br>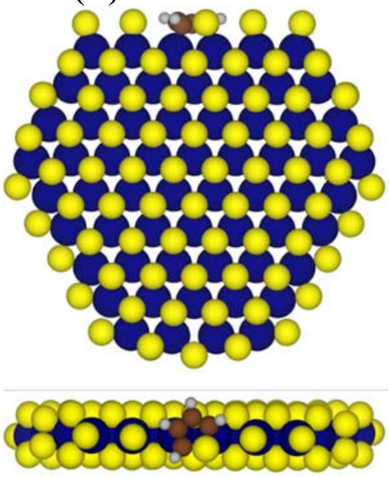<br>**Notes: Most stable thiophene adsorption on the mid-CUS of the Mo-edge of a particle with six (five) Mo (S) atoms. Note that this configuration displaces all S atoms on the edge to shift to an "on top" configuration with respect to Mo atoms on the edge.**

(v) BE = -1.45 eV

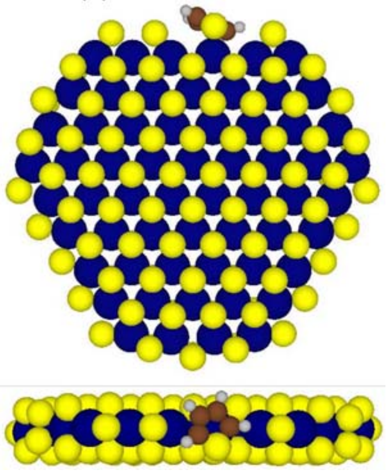

Notes: The binding of thiophene on A-M site (atop Mo) on two contiguous vacancies.

(vi) BE = -0.04 eV (-0.97 eV)

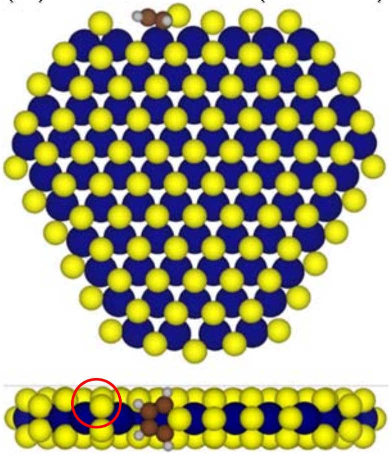

Notes: thiophene adsorption on the A-M site with S-dimer formed on the corner. Note that, with respect to an endge with a single CUS site, the binding energy is -0.04 eV; with respect to rearranged edge, the binding energy is -0.97 eV.

(vii) BE = -1.1 eV

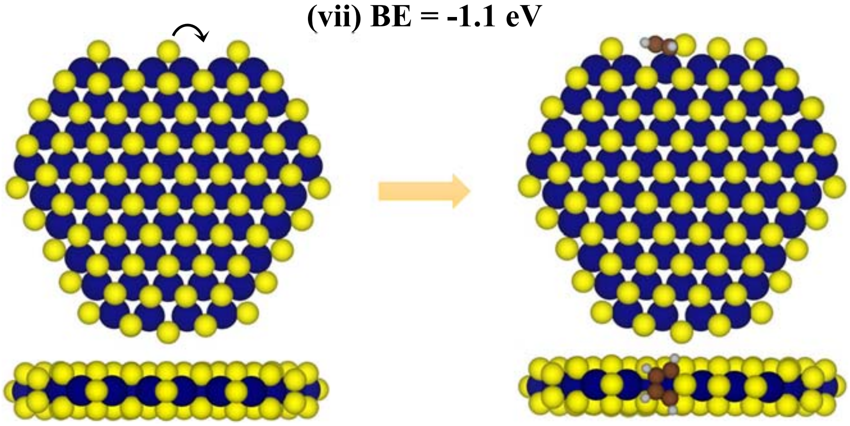

Notes: This binding energy of thiophene adsorption on adjacent sites is calculated relative to starting from two vacancies on A site and an S on the M site. The arrow points how the S atom on the M site moves to the neighboring A site to create the double vacancy.

Supplementary Table 2

The rearrangement energy (ΔE) for sulfur dimerization (in the absence of thiophene adsorption). Here, the rearrangement energy, ΔE, is given by: $\Delta E = E_{dimer} - E_{50\%S\text{-decorated edge}}$, where E_{dimer} is the energy of the nanocluster with dimers, $E_{50\%S\text{-decorated edge}}$ is the energy of nanoclusters with 50% S-decorated M-edge. In each case the dimer is indicated by a red circle in the edge (side) view. The reference for each case is a 50% S-decorated Mo-edge.

**ΔE = 1.84 eV**<br>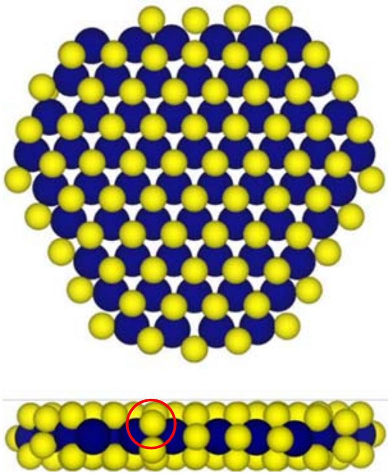<br>**Notes: The relative energy between 50% Mo-edge with monomers and its rearrangement to form an S-dimer in the A site to form vacancy on the C site.**	**ΔE = 0.85 eV**<br>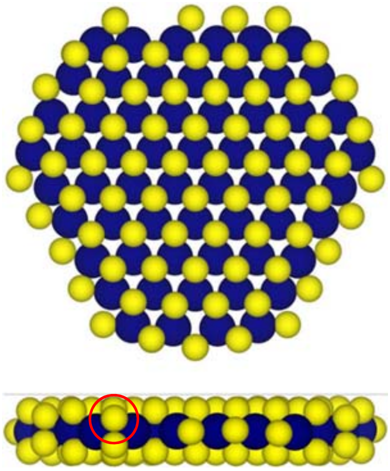<br>**Notes: The relative energy between 50% Mo-edge with monomers and its rearrangement to form an S-dimer in the C site to form a vacancy in the A site.**
**ΔE = 0.79 eV**<br>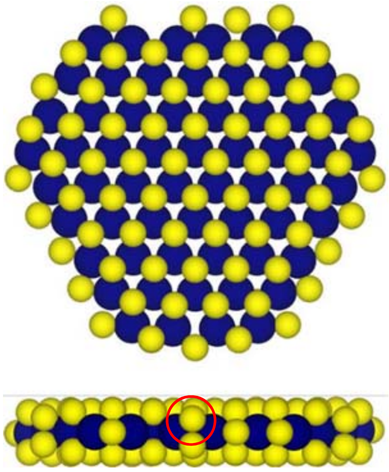<br>**Notes: The relative energy between 50% Mo-edge with monomers and its rearrangement to form an S-dimer in the M site to form vacancy on the A site.**	**ΔE = 0.74 eV**<br>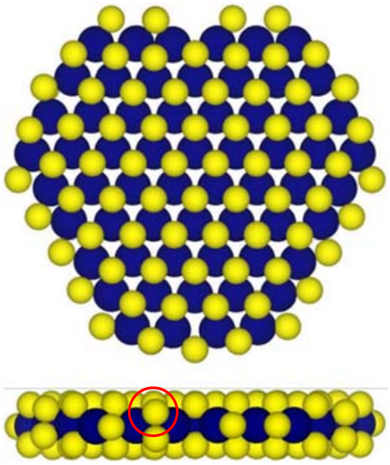<br>**Notes: The relative energy between 50% Mo-edge with monomers and its rearrangement to form an S-dimer in the A site to form vacancy on the M site.**

Supplementary Table 3

Energetics and structures for sequential and concerted S-dimerization. For sequential S-dimerization, the barrier is 2.1 eV. The subsequent thiophene adsorption is barrierless. The concerted step of simultaneous adsorption of thiophene and S dimerization has a barrier of 1.28 eV. Energetics of S-dimerization and concerted thiophene adsorption with S-dimerization. All values are in eV. The models are all periodic.

**Energetics of S-dimerization: Note that the starting state is representative of an M vacancy on the edge.**		
	**E_a = 2.11**	**ΔE = 1.78 eV**
**Initial state**	**Transition state**	**Final state**
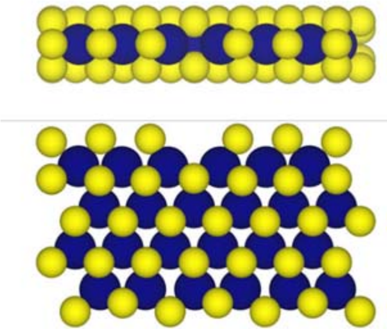	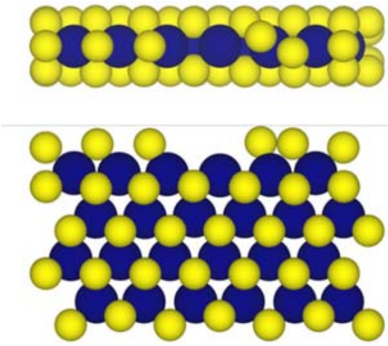	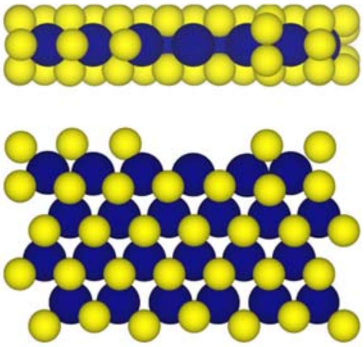
**Energetics of concerted thiophene adsorption and S-dimerization.**		
	**E_a = 1.28**	**ΔE = -0.14 eV**
**Initial state**	**Transition state**	**Final state**
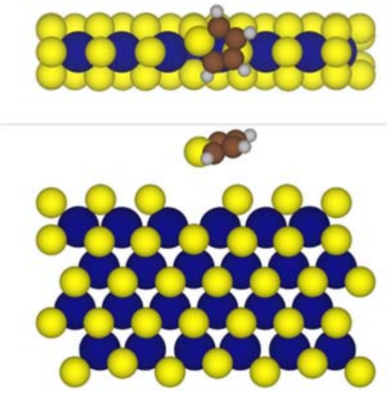	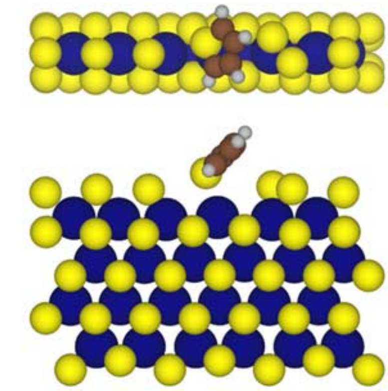	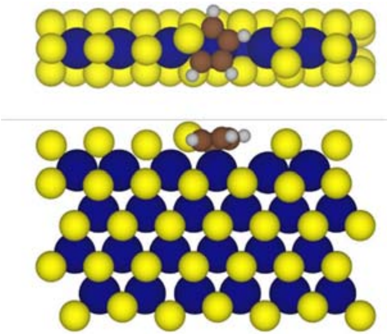
